# A school intervention for mental health literacy in adolescents: effects of a non-randomized cluster controlled trial

**DOI:** 10.1186/1471-2458-13-873

**Published:** 2013-09-23

**Authors:** Ingunn Skre, Oddgeir Friborg, Camilla Breivik, Lars Inge Johnsen, Yngvild Arnesen, Catharina Elisabeth Arfwedson Wang

**Affiliations:** 1 Department of Psychology, Faculty of Health Sciences, University of Tromsø, Tromsø, Norway; 2 Psychiatric Research Centre, University Hospital of North Norway, Tromsø, Norway; 3 Clinic for Child and Adolescent Mental Health, University Hospital of North Norway, Tromsø, Norway; 4 Family Health Services, Mandal Municipality, Mandal, Norway

**Keywords:** Adolescents, Universal intervention, Mental health literacy, Prejudice

## Abstract

**Background:**

“Mental health for everyone” is a school program for mental health literacy and prevention aimed at secondary schools (13–15 yrs). The main aim was to investigate whether mental health literacy, could be improved by a 3-days universal education programme by: a) improving naming of symptom profiles of mental disorder, b) reducing prejudiced beliefs, and c) improving knowledge about where to seek help for mental health problems. A secondary aim was to investigate whether adolescent sex and age influenced the above mentioned variables. A third aim was to investigate whether prejudiced beliefs influenced knowledge about available help.

**Method:**

This non-randomized cluster controlled trial included 1070 adolescents (53.9% boys, M age14 yrs) from three schools in a Norwegian town. One school (n = 520) received the intervention, and two schools (n = 550) formed the control group. Pre-test and follow-up were three months apart. Linear mixed models and generalized estimating equations models were employed for analysis.

**Results:**

Mental health literacy improved contingent on the intervention, and there was a shift towards suggesting primary health care as a place to seek help. Those with more prejudiced beleifs did not suggest places to seek help for mental health problems. Generally, girls and older adolescents recognized symptom profiles better and had lower levels of prejudiced beliefs.

**Conclusions:**

A low cost general school program may improve mental health literacy in adolescents. Gender specific programs and attention to the age and maturity of the students should be considered when mental health literacy programmes are designed and tried out. Prejudice should be addressed before imparting information about mental health issues.

## Background

Public knowledge and beliefs about mental disorders, also termed mental health literacy, may be crucial to the early recognition of mental health problems, and to the seeking and acceptance of mental health care [1,2]. Most mental disorders show their first signs, and increase in prevalence, from childhood through adolescence [3]. This is true both for anxiety, depression, schizophrenia [3] and eating disorders [4]. Universal health promotion/ prevention programmes are aimed at all members of a population or cohort, in contrast to targeted programmes, that are aimed at specific risk groups, or sub-groups [5]. The school is the obvious arena for universal programmes [6,7]. A considerable research literature exists on health promotion programmes aimed at schools, mainly from English language countries [5]. Norwegian health authorities have recommended a series of programmes for use in schools [8]. The efficacy of some programmes in Norwegian samples has been studied [9,10]. The purpose of the present study was to examine the possible effect of the universal school programme “Mental health for everyone” [11].

### Mental health literacy

Jorm [1,2] defines mental health literacy as: a) recognition of mental disorder, knowledge and belief about b) risk factors and causes, as c) self-help interventions and d) professional help available, as e) attitudes which facilitates recognition and appropriate help seeking, and finally as f) knowledge about how to seek mental health information. According to this definition, there should be a direct connection between knowledge about and attitudes towards mental health problems, and the ability to both recognise symptoms of mental disorder, and to seek help appropriately.

#### Recognition of mental disorder

Recognition of mental disorder has been operationalized [12] as the ability to identify and name a mental disorder based on a written case vignette. Studies have reported conflicting results for the identification of disorders from such vignettes. Jorm and colleagues [12] found that 40% of an adult Australian sample identified depression and 30% identified schizophrenia. Lauber and colleagues [13] found that 40% of a Swiss sample aged 16–76, identified depression and 75% identified schizophrenia. The highest level of case recognition for depression (81%) was reported in rural residents in Queensland, Australia by Bartlett and colleagues [14]. Contrasting, Suhail [15] found that only 20% percent of Pakistani people were able to identify depression, and even less (5%) were able to identify psychosis from a written vignette, and furthermore that level of education was a predictor of correct identification. In young Australian respondents, the ability to identify depression (50%) has been reported to be better than the ability to identify psychosis (25%) [16]. US adolescents had problems recognising both a depression vignette (42% correct) and an anxiety vignette (28% correct) as a mental health problem [17]. Women of all age groups are generally better than males in identifying depression [16,18]. Consequently, the ability to recognise a mental disorder seems to vary with gender, age, country, and level of education. Only a few studies report symptom knowledge in adolescents. Consequently, more research is needed on this age group.

#### Prejudiced beliefs

Prejudice, or stigma, is a complex construct, and can theoretically be broken down into cognitive, affective and behavioural domains. The cognitive part refers to stereotyped knowledge and beliefs, the affective domain refers to negative affects like aversion, embarrassment, shame, fear and aggression, and the behavioural aspect refers to avoidance and isolation of, and to discrimination against the object of prejudice [19]. Sartorius [20] suggests that the stigma attached to mental illness is the main obstacle to provision of care for mental health problems. In a review of the literature, Rüsch and colleagues [21] suggested that both self-stigma and fear of stigma are barriers against using health services. Self-stigma refers to perceiving ones symptoms as signs of being weak, stupid, lazy or even evil, rather than understanding that one has a mental health problem that could be helped by health professionals.

In a study of Norwegian high-school students, young men had higher levels of stigmatizing attitudes towards mental health problems, compared to young women [9]. In a Canadian study of adults, stigmatizing attitudes about depressed individuals correlated negatively with identification of a depressive case vignette [22]. According to Thornicroft and colleagues [23], stigma refers to problems of ignorance, prejudice and discrimination, in the way that lack of knowledge and prejudiced attitudes towards people with mental illness results in discrimination. Kroger and Marcia [24] connect stereotypes to the earlier and immature developmental stages of identity formation in adolescence, thus age, or cognitive and emotional maturity, may also be a crucial factor in studying prejudice.

Based on theories for understanding mental health stigma, Corrigan and colleagues [25] suggest three levels of programs for stigma change: public programs, programs targeted on specific groups, and individualized programs for coping with self-stigma.

#### The Norwegian health care system for mental health

In Norway, adolescents have easy access to the primary health care system through the school nurse, and all have a designated general practitioner (GP). All schools have school counsellors. The educational psychological services are only available through referral, and deal mainly with educational problems. Some communities, like the town in which the present study was performed, have low threshold psychological counselling services for youth [26]. For more severe emotional and behavioural problems there are specialist mental health services available, to which you have to be referred by your GP. Thus, the GP, the school nurse, the school counsellor and low threshold youth counselling services represent the primary level of care in Norway. The GP and the school counsellor are the “gate keepers” to the specialist mental health services, and to the educational psychological services, respectively. Primary health care is free of charge, except the GP, where you have to pay a small fee. Out-patient specialist mental health care is free of charge for citizens below age 18.

Health service use was reported by the total cohort of North Norwegian adolescents aged 15–16, and 25% of these had visited the school nurse, approximately 50% had visited their GP, and nearly 6% had seen a psychologist, or a psychiatrist (specialist services) in the preceding year [27]. Turi and colleagues [27] also found that girls utilised the school nurse and GP more than boys, while no gender difference was found for the use of specialist services.

#### The relationship between mental health knowledge, prejudice and help-seeking

Recognition of symptoms and lack of fear of stigma may be crucial to adequate help-seeking for mental health issues. Stigmatization, self-stigmatization, embarrassment and shame are often seen in connection to mental disorders.

After a mental health teaching programme, adolescents showed more understanding and empathy, and used less negative expressions to describe mental health problems [28], demonstrating a path between knowledge and prejudice. Depressed subjects reported feeling embarrassed about seeking professional help for them-selves, and had negative expectancies about how other people would react to them [29]. Depressed subjects reported lower probability for seeking help from professional sources, compared to non-depressed subjects, probably at least partly because of self-stigmatization [29]. Thus sufferers from mental health problems may hinder themselves from proper help-seeking.

Knowledge should be vital to help-seeking. If you do not recognise the symptoms and understand that they are signs of a health problem, the probability is low for consulting health professionals. Olsson and Kennedy [17] found that those who recognized a disorder as a mental health problem had much higher probability to suggest seeking help for the problem, than those who did not identify the health problem.

Gender differences in these aspects of mental health literacy have also been demonstrated. In their study of young Australians, Cotton and colleagues [16] found that roughly half of both women and men suggested seeing a doctor or a specialist for symptoms of depression, while two thirds of the young women, but only half of the young men, suggested seeing a doctor or a specialist for the treatment of psychosis. Burns and Rapee [18] found that girls were more likely to suggest seeing a counsellor for depressive symptoms, while both genders endorsed other health services equally.

### School programmes for mental health literacy

Several universal school programmes for early prevention of behavioural and emotional problems are available. These programmes are more or less based on psycho-educational and experiential principles. Some of the programmes available in Norway are translated and adapted, while others are developed in Norway, like the program presented here, “Mental health for everyone” [11], aimed at students in secondary schools, 13–15 year olds.

#### Mental health for everyone

The universal mental health promotion program “Mental health for everyone" is available online in Norwegian, from a governmental website, free of charge [11]. Both an English language and an Arabic language translation are available from the Norwegian Council of Mental Health. The program is based on Antonovsky’s theory of salutogenesis [30]. Based in positive psychology, the salutogenetic perspective aims at health promotion through empowerment [31]. The basic principle of empowerment health education [32] is to engage participants in group experiences and group dialogues, in order to stimulate control and beliefs in the ability to change their own behaviour. Meta-analyses of the effectiveness of health promotion programmes have confirmed that programmes focusing on positive mental health, and engaging students in practical tasks and activities are more likely to succeed, compared to programmes dominated by lectures and delivering of knowledge [5]. The aims of “Mental health for everyone” are fourfold: 1) To contribute to the prevention of mental disease, 2) To challenge the attitudes and prejudices against mental health problems and the mentally ill, 3) To contribute to openness and confidence about mental health issues, 4) To impart knowledge about mental health services and availability of help for mental health problems.

The available material for teachers [11] includes three packages with student tasks and video-material, one for each grade (8th to 10th) of Norwegian secondary school. Each package has a basic theme: For the 8th grade the themes are *Self-awareness and Identity*; for the 9th, *Being different, and Loneliness*; and for the 10th grade *Fear of the Unknown.* The pedagogy of “Mental health for everyone” was chosen and organized so as to actively engage and include all students. Consequently, the themes and tasks are meant to be varied and engaging, and to capture the attention of the students throughout three consecutive school days. The pedagogy includes individual tasks, group tasks and plenary sessions, and illustrating video material is included. When implementing the school package for the first time, schools are advised to use the 8th grade programme for all levels, since the programmes build upon each other, and this was done in the present study. The theme Self-awareness and Identity for the 8th grade have three subsidiary themes: *Well-being, mental health problems, and mental disorders*. For these themes the teachers are free to choose among a variety of tasks, whichever he or she finds most suiting for their class. All tasks are relatively short, and intend to catch attention and inspire to reflect around the chosen theme. Examples of individual student tasks for the theme *identity and well-being* are: 1) to make a play-list of her or his favourite music, or 2) to bring dispensable items symbolizing favourite themes (i.e. movie or concert tickets, postcards) to school and use them for a collage representing him or herself. An example of a plenary task is to arrange a catwalk where the commentator has been secretly instructed to only comment on the positive personal and inner qualities of the “model”, and not on looks; and the audience has been instructed to cheer, no matter what happens. After the show the class discussion is aimed at the experience of valuing inner rather than surface qualities.

Lectures on the most common or well-known mental disorders are given for each level in the programme, and in the 8th grade anxiety, depression, eating disorders and schizophrenia are presented. In consecutive years other common disorders, like ADHD, bipolar disorder, psychosomatic disorders and problems with self-harm and suicide, are presented. Each year, practical information about where and how to find help is included for all levels. However, the main focus is on positive mental health, and only one lesson in the three-day programme is aimed at information about specific mental disorders.

### Aims

The primary aim of this study was to investigate whether adolescent mental health literacy, could be improved by means of a universal education programme by:

a) improving naming of symptom profiles of mental disorder,

b) reducing prejudiced beliefs about mental illness,

and

c) improving knowledge about where to seek help for mental health problems.

A second aim was to investigate whether adolescent sex and age influence mental health literacy.

The third aim was to investigate whether prejudiced beliefs about mental illness affected knowledge about available help.

## Methods

### Socio-economic and cultural setting of the study

Norway is an egalitarian country with rather high standards of living and with small socio-economic differences. The Norwegian compulsory school system is financed and regulated by the authorities, and only 2% of Norwegian children and adolescents attend private schools. Compulsory primary school covers grades 1st (age 6) through 7th (age 12), and secondary school covers grade 8th (age 13) through 10th (age 15). The study was conducted in 2005 in a North Norwegian town with a population of 70,000. North Norway has a multi-ethnic population with a Norwegian majority, an indigenous Sami minority of about 10%, and other ethnic groups representing around 5–10% of the population. The Sami population is well integrated, and has equal education and living standards as the majority population. In a recent population based study from North Norway, no differences in socio-economic status or internalisation symptoms were found between indigenous Sami and ethnic Norwegian adolescents [33]. The participating schools were situated in residential and suburban areas. These schools scored slightly above the national average on standardized academic tests when such results were available from 2008 [34].

### Design

**Figure 1:**
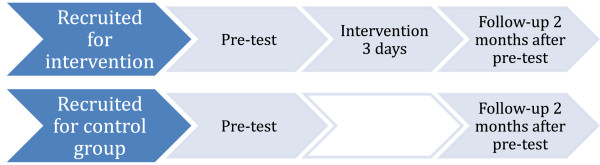
The design of the study.

The study was a cluster controlled trial, since randomization was not possible at the individual level. The design is presented in Figure 1. The entire school was assigned to either the intervention, or to the control group. A pre-test was performed by questionnaire in both control and intervention schools. The three-day intervention followed immediately after the pre-test. The follow-up was performed in both intervention and control schools two months after the pre-test. Thus, two measurements were performed in all schools.

### Procedure

#### Recruitment

Four secondary schools with a total of 1500 students were invited. One school with approximately 400 students declined to participate. The remaining three schools (1100 students) agreed to participate in the study. The largest school gave consent for the school to participate as an intervention school. The two remaining schools were assigned to the control group. The researchers had to yield to the fixed school schedules, and the principals of the schools decided when the data collection and intervention could be performed. The data was collected three months earlier in the school year in the intervention school than in the control schools.

#### Briefing of teachers prior to the intervention

Three of the authors (CB, LIJ and YA) performed the data collection, and also briefed the teachers at the intervention school one month prior to the intervention. Before the briefing, the teachers received the manual for “Mental health for everyone”, the teacher’s guide, and written information about the four mental disorders depression, anxiety, eating disorders and schizophrenia. The aim of the meeting was to present the school package, to answer any questions, and to inform about the research project. The participants were also given one of the tasks in the manual with the purpose to give the teachers a “hands on” experience with the programme, and to inspire for discussion. Emphasis was given to the importance of respect for the personal integrity of the students when approaching the theme mental health. The teachers were informed about the structure of the mental health care system and where and how to seek help. Teachers at each grade subsequently met in groups with the authors to plan for the three-day intervention.

#### Implementation and mental health literacy lectures

The major part of the three school days was spent engaging the students in tasks and activities chosen from the manual by their class teachers. The three co-authors were at the disposal of the teachers for imparting the information about mental disorders, knowledge about mental health problems and about the available mental health services. For each class, one school lesson was dedicated to information about the clinical picture, epidemiology and treatment of anxiety, depression, eating disorders and schizophrenia.

In 17 of the 23 classes, the teachers asked the authors to lecture about the mental disorders and the available help system. Thus in almost ¾ of the classes this part of the curriculum was taught in a more or less uniform manner by the three co-authors, who were graduate students in clinical psychology. In the remaining ¼ the class teachers taught these subjects after being briefed by the same three authors.

#### Measurements and anonymity

At the intervention school the pre-test was performed in the beginning of January, in the morning of the same day the intervention started. The follow-up was performed in March, two months after the intervention. Teachers distributed and collected the questionnaires during the same school lesson.

In school B, the pre-test was performed in April, and the follow-up was performed in June. In school C the corresponding data were collected in June (end of academic year) and August (beginning of the next academic year). Consequently, the data collection of both the pre-test and the follow-up in the intervention school A were performed 3 and 5 months earlier in the year, than in the control school B and C, respectively. In all schools the interval between pre-test and follow-up was 2 months. The authors administrated the questionnaires in the control schools.

For identification and anonymization, students were given ID-numbers. The list linking names and numbers was kept by the teacher. The ID-number followed the pupil through the pre-test and the follow-up. After the follow-up, the teachers were instructed to destroy the list linking names and numbers, to safeguard the anonymity of the respondees.

### Ethics

The research project was approved by the Regional committee for ethics in medicine in Health region Northern-Norway. Informed consent was obtained by sending an information letter to all parents and students, informing about the study and that participation was voluntary. The majority of the students were minors (below age 16) according to Norwegian health law. The response procedure guaranteed total anonymity for the individual respondents. Informed consent was considered appropriate for this study, by the ethical committee. Respondents were informed that participation was voluntary and that they could refrain from answering any question.

### Instruments

A 66-item questionnaire, of which 7 questions were open ended, was employed. *Demographic variables* collected were age, sex, school and grade, and a constructed ID-number.

#### Measurement of mental health literacy

*Symptom profile recognition.* Four symptom profiles were presented in the questionnaire, and participants were asked open-ended questions to name the disorder (correct answers in parenthesis): “*Name the disorder characterized by lack of energy, problems concentrating*, *lack of initiative, and by sadness and withdrawal from social activities.”* (Depression)*, “Name the disorder characterized by delusions, disturbed thoughts, and strange sensual experiences.”* (Psychosis/ schizophrenia)*.” Name the disorder characterized by a feeling of uneasiness, feeling of panic, of fear and rapid heart-beat.”* (Anxiety), and, finally,*” Name the disorder characterized by fear of weight gain, by low food intake and extremely low weight.”* (Anorexia nervosa/ eating disorder)*.* No response alternatives were offered.

For each correct answer, 1 point was given, and the mean of the four answers constituted the scale *Knowledge about mental disorders*, with a minimum value 0 and a maximum value 1. The internal consistency of the scale was fairly high, Cronbach’s alfa = .80.

*Prejudiced beliefs.* Items measuring prejudiced beliefs towards mental illness were constructed by the authors in collaboration with the Norwegian Council for Mental Health. The scale consisted of four statements, considered to be representative of commonly encountered prejudiced beliefs. The statements were rated on a 5-point Likert scale ranging from *“disagree completely”* (score 0) to *“totally agree”* (score 4). The first two statements: *“All who have a mental illness should be committed to a mental hospital.”* and *“All schizophrenics are violent.”* were inspired by a debate in Norway, following a tragedy when a newly discharged psychiatric patient stabbed and killed a fellow passenger and wounded several others on a tram in Oslo. The last two statements: “*Those who become mentally ill are weak people.”* and *“You must really be in trouble if you see a psychologist”,* were inspired by comments from adolescents participating in a pilot to the present study [35].

Exploratory factor analysis for these four items was performed, and only one principal component emerged (Eigenvalue 1.8). The four items loaded fairly equally on the component (.61 - .71). The mean score of these four items was calculated, constituting the scale *Prejudiced beliefs,* ranging from 0.0 (no prejudiced beliefs) to 4.0 (maximum prejudiced beliefs)*.* In the present sample Cronbach’s α for the scale was .56 at pre-test and .76 at follow-up. Although the internal consistency at pre-test was questionable, the α at follow-up was acceptable. Furthermore, the one principal component solution for these four items indicates that they constitute a shared construct. Since prejudice is a complex construct, as described in the introduction a very high internal consistency was not to be expected. A possibly adding more items with similar content would not guarantee an increased α either. This was demonstrated by Andersson and colleagues [8], who adopted this scale. They added two items: “*It is difficult to talk to people with mental health problems”* and *“ADHD is caused by bad manners”*, and obtained a similar one principal component solution, similar loadings, and an α of .78 in a representative sample of Norwegian 16–18 year old high-school students.

*Knowledge about where to seek help for mental health problems.* The questionnaire included open-ended questions about the mental health care system. The answers to the item: *“There are places where you can seek help for mental health problems. Write down the places that you know of.”* were employed in the present study. No response alternatives were given, so the adolescents had to formulate their own answers. Answers were sorted into four categories. All who left this question unanswered, or who answered “I don’t know” were placed in the category *“No answer given”*. All who mentioned parents, siblings, other family members, friends, self-help, the Internet, or other non-professional sources of help, were placed in the category *“Home, self-help, internet”.* All who mentioned GP, school nurse, school counsellor or low threshold counselling service, were placed in the category *“Primary care”.* Finally, the category *“Specialist care”* included all answers mentioning health care requiring a referral from the primary health care system: The Child and Adolescent Mental Health Out-patient Clinic, or Hospital, psychiatrists, psychologist or educational psychologist.

### Treatment of missing data

For cross-sectional analyses at pre-test and post-test, data from all students who returned their questionnaire were included. Missing data when calculating scale scores were treated as follows: On the *Symptom profile recognition* and *Prejudiced beliefs* scales, only one missing item out of four was allowed, and mean score were calculated based on the items that were answered.

### Statistical analyses

IBM SPSS version 19.0.0 was employed for all statistical analyses. Simple between group differences were examined using chi-square tests for dichotomous and analysis of variance (ANOVA) for continuous variables. Analyses of change (pre-test to follow up) were conducted using linear mixed model regression analyses for continuous outcome variables, and generalized estimating equation (GEE) regression models for dichotomous outcome variables. A logit link function and a binomial distribution were specified for the latter. Robust estimation of standard errors (Huber-White correction) was used.

## Results

### Participants and attrition

**Figure 2:**
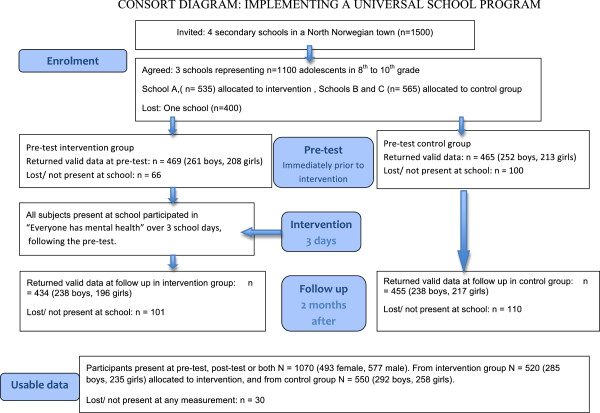
Inclusion and attrition of participants shown in accordance with the consort guidelines.

The recruitment and attrition of participants is presented according to the Consort guidelines in Figure 2, and distribution of those who returned usable data on any measurement are presented in Table 1.

**Table 1:** Subjects returning usable data at pre-test, follow-up, or both, by sex and group (N = 1070)

	** n (%)**	** Sex**	** n (%)**
Intervention group	520 (48.6)	Girls	235 (45.2)
		Boys	285 (54.8)
Control group	550 (51.4)	Girls	258 (46.9)
		Boys	292 (53.1)
Total sample	1070 (100.0)	Girls	493 (46.1)
		Boys	577 (53.9)

Out of 4 schools (n = 1500), three schools (73% of students) agreed to participate (n = 1100). A total of 1070 students (97%) out of the eligible 1100 participated in the pre-test, the follow-up, or both (Figure 2). All 520 students at the largest school (A) received the intervention, while 550 students from the two smaller schools (B and C) formed the control group. Out of the 1070 that returned usable data, the participation rate was significantly higher in the intervention group (90%) compared to the control group (85%) (Χ^2^_1df_ = 7.68, p < .005). There were more boys (55%) than girls (45%) in both intervention and control group.

All students present at school at the day of the pre-test were included. The pre-test was done in the first lesson, followed by the 3-day intervention for those assigned to the intervention group. Thus, there was no attrition from the intervention group from pre-test to intervention. At follow-up, usable data was obtained from 889 (83.1%) out of the 1070 students. The participation rate at follow-up was nearly equal in the intervention (83.5%) and control (82.7%) group. At follow-up 476 (53.5%) boys and 413 (46.5%) girls participated in the study. Out of the 1070 that participated at either pre-test or follow-up, or both, 834 (77.9%) returned complete data at both occasions, while 236 (22.1%) returned complete data at only one occasion. The participation rate for both measurements was relatively equal in both groups, with 399 (76.7%) returning complete data at both pre-test and follow-up in the intervention group, and 445 (80.9%) in the control group (NS).

No statistically significant differences on outcome variables were found between those who had complete data at pre-test and missing data at follow-up, but there was a tendency that those who had missing data at follow-up had more prejudiced beliefs (F = 3.521, p = .06). On the other hand, those who did not participate at pre-test, but who returned valid data at follow-up, had less symptom profile knowledge (F = 16.15, p < .0001) and more prejudiced beliefs (F = 8.27, p = .004) compared to those who returned valid data on both occasions.

#### Sample characteristics

In the intervention group, the age range was 12–16 years (*M* = 14.06, *SD* = .85), while the age range in the control group was 13–17 years (*M* = 14.29, *SD* =. 82). The control group was significantly older than the intervention group by 3 months (ANOVA, F = 17.08, p < .001). This age difference reflects that the data was collected later in the school year in the control schools than in the intervention school.

Table 2 shows the numbers of valid and missing cases for the outcome variables at pre-test and follow-up. At pre-test 87% of the sample returned usable data, while 83% delivered usable data at follow-up.

**Table 2:** Valid data for dependent variables at pre-test and follow-up

**Variable**	**Pre-test**								**Follow-up**							
	**Cases**						**Values**		**Cases**						**Values**	
	**Valid**		**Missing**		**Total**		**Freq.**	**Value**	**Valid**		**Missing**		**Total**		**Freq.**	**Value**
	**N**	**%**	**N**	**%**	**N**	**%**	**n (%)**	**Mean (SD)**	**N**	**%**	**N**	**%**	**N**	**%**	**n (%)**	**Mean (SD)**
Depression profile	934	87.3	136	12.7	1070	100	264 (28.3)	--	890	83.2	180	16.8	1070	100	367 (41.2)	
Schizophrenia profile	934	87.3	136	12.7	1070	100	224 (24.0)	--	889	83.1	181	16.9	1070	100	350 (39.4)	
Anxiety profile	935	87.4	135	12.6	1070	100	116 (12.4)	--	890	83.2	180	16.8	1070	100	258 (29.0)	
Eating disorder profile	935	87.4	135	12.6	1070	100	619 (66.2)	--	889	83.1	181	16.9	1070	100	590 (66.4)	
Mean symptom profile recognition	934	87.3	136	12.7	1070	100	--	0.33 (0.29)	889	83.1	181	16.9	1070	100	--	0.44 (0.38)
Mean prejudiced beliefs	930	86.9	140	13.1	1070	100	--	2.3 (0.77)	876	81.9	194	19.1	1070	100	--	2.2 (0.85)
Places for help	928	86.7	142	13.3	1070	100			884	82.6	186	17.3	1070	100		
No places mentioned							222 (23.7)								171 (20.3)	
Home/friends/internet							59 (6.3)								82 (9.7)	
Primary health care							211 (22.6)								268 (31.7)	
Specialist health care							443 (47.4)								324 (38.3)	

#### Identification of symptom profiles

First, correct identification of the four symptom profiles was studied (Table 3). At pre-test *eating disorder* was identified by more than half of the students in both control group and intervention group. The second most recognized disorders at pre-test were *depression* and *schizophrenia,* identified by one fifth to one third of the students. *Anxiety* disorder was only identified by 12-13% of students at pre-test. Correct naming of *schizophrenia, depression and eating disorder* was significantly more common in the intervention group at pre-test, while the *anxiety* disorder profile was equally unrecognized in intervention and control group.

**Table 3:** Correct identification of symptom profiles, by group, at pre-test and follow-up

**Symptom profile**		**Pre-test**			**Follow-up**		
	**Condition**	**N**	**n (%) correct**	**Χ**^**2 **^**(1 df)**	**N**	**n (%) correct**	**Χ**^**2 **^**(1 df)**
		**935**		**p**	**890**		**p**
Schizophrenia	Intervention	469	138 (29.4)	15.30	433	271 (62.6)	190.62
	Control	465	86 (18.5)	<.0001	456	79 (17.3)	<.0001
Depression	Intervention	468	162 (34.6)	18.65	434	267 (61.5)	143.84
	Control	466	102 (21.9)	<.0001	456	100 (21.9)	<.0001
Anxiety	Intervention	469	57 (12.2)	.060	434	199 (45.9)	117.02
	Control	466	59 (12.7)	n.s.	456	59 (12.9)	<.0001
Eating disorder	Intervention	469	340 (72.5)	16.65	434	333 (76.6)	40.79
	Control	466	279 (59.9)	<.0001	455	257 (56.5)	<.0001

Table 3 also shows symptom profile recognition at follow-up. For all the four disorders, the proportions of correct profile recognition remained the same at pre-test and follow up in the control group. For all symptom profiles the differences between intervention group and control group were larger at follow-up. This is shown by higher proportions for correct identification in the intervention group, and by substantial increase in the Χ^2^-statistics for the differences between intervention and control group.

Gender differences for the recognition of almost all disorders were found at the pre-test (data not shown). Girls identified symptom profiles better than boys, and the gender difference was significant for depression (39% vs. 20%, Χ^2^ (1df) = 42.6, p < .0001), anxiety disorder (16% vs. 9%, Χ^2^ (1df) = 10.0, p < .001) and eating disorder (78% vs. 56%, Χ^2^ (1df) = 49.3, p < .0001). There was however no significant gender difference for the identification of schizophrenia (27% vs 22%, n.s.).

Mean scores for correct *symptom profile recognition* were calculated. To study the possible impact of the intervention on *symptom profile recognition,* mixed models analyses were performed (Table 4 and Table 5). For all models, the variables *sex (girl = 0, boy = 1), age, school (A, B or C), grade (8th, 9th or 10th), group (intervention vs control), time (pre-test vs post-test) and group x time (the interaction between group status and time)* were entered as independent variables. In Table 4, descriptive statistics of mean scores for symptom profile recognition at pre-test and follow-up, as calculated by Generalized mixed model anaysis are presented. In the control group a mean score of .31 reflects that the control school adolescents recognized slightly less than one third of the symptom profiles correctly at both pre-test. The score .38 reflects that the intervention group identified somewhat more than one third of the profiles correctly at pre-test.

**Table 4:** Symptom profile recognition at pre-test and follow-up and change over time, estimated by generalized linear models

**Dependent variable**	**Control group**				**Intervention group**			
	**Pre-test**	**Follow-up**	**Change**		**Pre-test**	**Follow-up**	**Change**	
			**Pre-test – Follow-up**				**Pre-test – Follow-up**	
	**Mean**	**Mean**	**Mean difference**	**p**	**Mean**	**Mean**	**Mean difference**	**p**
	**(95% CI)**	**(95% CI)**	**(95% CI)**		**(95% CI)**	**(95% CI)**	**(95% CI)**	
Symptom profile recognition	0.31	0.31	0.00	.92	0.38	0.64	0.27	.0001
	(0.28 - 0.35)	(0.28 - 0.35)	(−0.03 - 0.03)		(0.35 - 0.41)	(0.61 - 0.67)	(0.24 - 0.30)	

**Table 5:** Generalized mixed models for symptom profile recognition

	***Symptom profile recognition***			
**Independent variables**^**1**^	**Beta**	**β**	***t***	***F***
Intercept	.31			
Group (0 = control, 1 = intervention)	.07	.22	3.25***	98.49***
Time (0 = pre-test, 1 = follow-up)	-.00	.00	0.14	155.96***
Group x Time	.27	.78	12.70***	161.19***
Age	*ns*			
Grade				4.03*
9th vs 10th	.02	.05	*ns*	
8th vs 10th	.11	.31	2.12*	
Sex (0 = female, 1 = male)	-.14	-.42	8.17***	66.76***

*Notes*. Scores: Beta-values represent percentage of correct symptom profile recognition (ex: .31 = 31% correct). β = Standardized beta-values.

^1^ School was controlled for, but did not contribute significantly in the model.

* *p* < .05, ** *p* < .01, *** *p* < .001. F-values are based on Type III sum-of-squares and corrected for non-orthogonality. As t-tests are uncorrected, *F*-tests are relied on for significance testing of effects.

Table 4 also shows that there was no change from pre-test to follow-up in the control group, while the mean recognition of symptom profiles nearly doubled in the intervention group (p < .0001).

The model for change over time in *symptom profile recognition* is presented in Table 5. School was controlled for, but showed no effect, and the variable was thus removed from the model. The independent variable *group* shows the main effect of group membership, the *time* variable shows the effect of time passing between pre-test and follow-up, while the interaction term *group x time* can be interpreted as the effect of the intervention. Both the effects of *group* and *group x time* were statistically significant, and the interaction term had the strongest Beta-value. This means that the intervention had an independent and stronger effect than the initial group differences on the outcome variable. No significant effect of *age* was found. However, since *grade* and *age* are highly correlated, the moderate, but significant effect of increasing school grade from 8th to 10th should be read as an age effect. *Sex* had the second strongest effect on symptom profile recognition, in that girls recognized symptom profiles better than boys.

#### Prejudiced beliefs

As shown in Table 2, observed mean score for prejudiced beliefs was 2.3 (SD .77) at pre-test and 2.2 (SD .85) at follow-up. When prejudice was measured on a 5-point likert-scale from disagree completely (0) to agree completely (4) in a prejudiced statement, a score of 2 means that the respondents neither completely reject nor completely accept the prejudiced statements, while a score above 2 means that they tend to agree.

To study the possible impact of the intervention on *prejudiced beliefs,* mixed models analyses were performed (Table 6 and Table 7). For all models, the variables *sex* (girl = 0, boy = 1), *age, school* (A, B or C), *grade* (8th, 9th or 10th)*, group* (intervention vs control)*, time* (pre-test vs follow-up) and *group x time* (the effect of the intervention) were entered as independent variables.

**Table 6:** Descriptive and inferential statistics for prejudiced beliefs estimated by generalized linear models

**Dependent variable**	**Control group**				**Intervention group**			
	**Pre-test**	**Follow-up**	**Change**		**Pre-test**	**Follow-up**	**Change**	
			**Pre-test – Follow-up**				**Pre-test – Follow-up**	
	**Mean**	**Mean**	**Mean difference**	**p**	**Mean**	**Mean**	**Mean difference**	**p**
	**(95% CI)**	**(95% CI)**	**(95% CI)**		**(95% CI)**	**(95% CI)**	**(95% CI)**	
Prejudiced beliefs	2.38	2.28	−0.10	.02	2.21	1.92	−0.29	.0001
	(2.27 – 2.44)	(2.19 – 2.37)	(−0.15 - 0.05)		(2.14 – 2.28)	(1.84 – 1.99)	(−0.37 - -0.22)	

**Table 7:** Generalized linear models for prejudiced beliefs

	***Prejudiced beliefs***			
**Independent variables**^**1**^	**Beta**	**β**	***t***	***F***
Intercept	1.04			
Group (0 = control, 1 = intervention)	-.14	-.18	2.48*	22.86***
Time (0 = pre-test, 1 = follow-up)	-.07	-.09	1.89	44.82***
Group x Time	-.22	-.28	4.04***	16.03***
Age	*ns*			
Grade				5.78**
9th vs 10th	-.21	-.26	2.82**	
8th vs 10th	-.43	-.52	3.40***	
Sex (0 = female, 1 = male)	.35	.43	7.99***	63.82***

*Notes*. Prejudiced beliefs: Beta-values represent raw score value (range 1–5). β = Standardized beta-values.

^1^ School was controlled for, but did not contribute significantly in the model.

* p < .05, ** *p* < .01, *** *p* < .001. F-values are based on Type III sum-of-squares and corrected for non-orthogonality.

Since t-tests are uncorrected, *F*-tests are relied upon for significance testing of effects.

Table 6 shows that there was a small decline in *prejudiced beliefs* in the control group (p < .02), and a marked decline from pre-test to follow-up in the intervention group (p < .0001).

The model for change over time in *prejudiced beliefs* is presented in Table 7. The independent variables entered in the model were the same as for symptom recognition. School was controlled for, but showed no effect, and the variable was thus removed from the model.

Both *group, time* and *group x time* contributed significantly to reduction in prejudiced beliefs from pre-test to follow-up, meaning that both the difference between control and intervention group, the passing of time, and being exposed to the intervention contributed to change in *prejudiced beliefs*. As for the previous model, the effect of *age* was not significant, but probably covered by the effect of increasing *grade*. *Prejudice* decreased with increasing school grade. Finally, *sex* contributed separately to *prejudiced beliefs*. Boys held more prejudiced beliefs than girls.

#### Places to seek help for mental health problems

In Table 8, the distribution of mentioned places to seek help for mental health problems are presented. At pre-test more in the intervention group mentioned *specialist psychological or psychiatric health care*, and more in the control group left the open-ended question unanswered, i.e. *no place mentioned*. Otherwise the groups were equal at pre-test.

**Table 8:** Proportion of sample mentioning places for help for mental health problems, estimates from generalized estimating equation model

		**No place mentioned**		**Home, self-help, internet**		**Primary health care**		**Specialist health care**	
Group	Time	Proportion	95% CI	Proportion	95% CI	Proportion	95% CI	Proportion	95% CI
Control	Pre-test	.24	(.20 - .28)	.08	(.05 - .10)	.22	(.18 - .26)	.42	(.37 - .46)
	Follow-up	.24	(.20 - .29)	.07	(.05 - .10)	.24	(.20 - .28)	.41	(.36 - .46)
Intervention	Pre-test	.18	(.15 - .22)	.05	(.03 - .07)	.23	(.19 - .27)	.54	(.49 - .58)
	Follow-up	.17	(.13 - .21)	.10	(.07 - .13)	.39	(.34 - .44)	.35	(.31 - .40)

At follow-up, the weight of answers in the intervention group seems to have shifted from *specialist health care* at pre-test, towards more mentioning of *primary health care* at follow-up. At follow-up there were also slightly more in the intervention group that mentioned *home, self-help, internet* as places to turn for help, compared to the control group. The distribution of answers in the control group remained unchanged from pre-test to post-test.

In Table 9 results from generalized estimating equation models for the spontaneously mentioning of places to seek help are presented. The four categories were treated as mutually exclusive, hence yielding four analyses. The categories were: 1) No places mentioned, 2) home, self-help, internet (e.g., parents, siblings, other family, friends, self-help, Internet) as a place to seek help, 3) primary health care (e.g., school nurse, GP, school counsellor, low-threshold community counselling service), and 4) specialist health care (i.e. clinical psychologist, psychiatrist, child and adolescent mental health service). Each category was compared with all the others.

**Table 9:** Generalized estimating equations models for spontaneously mentioned places to find help for mental health problems

	***No places mentioned***		***Home, self-help, internet***		***Primary health care***		***Specialist health care***	
Independent variables^1^	Exp(B)	95% CI	Exp(B)	95% CI	Exp(B)	95% CI	Exp(B)	95% CI
Intercept	.10***	.07 -.15	.09***	.09 - .16	.02***	.00 - .19	.62***	.50 - .77
Group (0 = control, 1 = intervention)	0.70*	.51 - .96	.61	.36 - 1.04	.96	.70 - 1.31	1.64***	1.27 - 2.12
Time (0 = pre-test, 1 = follow-up)	1.09	.86 - 1.40	.96	.62 - 1.47	1.07	.82 - 1.40	.92	.73 - 1.14
Group x Time	.94	.64 - 1.38	2.39**	1.25 - 4.56	1.75**	1.20 - 2.57	.50***	.36 - .69
Age	-	-	-	-	1.30***	1.12 - 1.50	-	-
Sex (0 = female, 1 = male)	-	-	-	-	.71**	.56 - .90	1.26*	1.02 - 1.55
Prejudiced beliefs (*m* = 2.24, *SD* = .81)	1.69***	1.46 - 1.96	-	-	.67***	.57 - .78	-	-

*Notes.* Grade and school were non-significant in all models. Variables reported as “-” were non-significant and was removed from the model, except for the intervention factors *group* and *time*.

* *p* < .05, ** *p* < .01, *** *p* < .001.

The variables of main interest were *group* (intervention vs control group)*, time* (pre-test vs follow-up), and the *group x time* interaction (effect of intervention). A significant interaction would support a differential change over time, and possibly confirm a hypothesis of intervention effects. The following variables were additionally included as covariates (but removed if not significant): *School, grade, age, gender* and *prejudiced beliefs*. The means referred below to are means calculated in post-hoc tests, and correspond to the proportions of sample answering in the four categories presented in Table 8 (M_intervention_ = mean proportion in intervention group, *M*_control_ = mean proportion in control group, M_pre_ = mean proportion at pre-test, M_follow-up_ = mean proportion at follow-up).

*No places mentioned* (leaving blank the question: *“There are places where you can seek help for mental health problems. Write down the places you know of”*): The *group* factor was significant (*M*_intervention_ = .18 and *M*_control_ = .24), but the interaction term was not. Thus, the intervention had no effect on this category, meaning that those who gave no answer at pre-test, continued to not answer this question after the intervention. The covariate *prejudiced beliefs* was significant, indicating that respondents with higher scores on *prejudice* more often chose the response category *n*o *places mentioned*

*Home, self-help, internet*: The *time x group* interaction was significant, indicating that change was contingent on the intervention. Post-hoc tests indicated no change from pre to post in the control group (*M* = .08 at both time points), while significantly more subjects mentioned this category following the intervention (*M*_*pre*_ = .05 to *M*_*follow-up*_ = .10, *p* < .001). None of the covariates affected the estimates.

*Primary health* care: Again, the *time x group* interaction was significant. There was no significant change in the control group (*M*_pre_ = .22, *M*_follow-up_ = .24), while significantly more subjects mentioned this category in the intervention group (*M*_pre_ = .23, *M*_follow-up_ = .39, *p* < .001). The estimates were adjusted for by the following significant covariates: older *age*, being a *female* and having *less prejudiced* beliefs increased the probability of mentioning primary health care like the GP, school nurse, school counsellor or other counselling services as places to turn to for help.

*Specialist health care*: Both *group* and the *group x time* interaction were significant, hence indicating an intervention effect. Again, the change in the control group was not significant (*M*_pre_ = .42, *M*_follow-up_ = .41), while the intervention group mentioned this category significantly less often following the intervention (*M*_pre_ = .54, *M*_follow-up_ = .35, *p* < .001). Gender was the only significant covaraite, indicating that boys mentioned specialist health care more often than girls.

## Discussion

The main finding in this study was that mental health literacy in terms of symptom profile identification, prejudiced beliefs, and knowledge about where to seek help, changed after a three-day universal school intervention aimed at secondary school students. Furthermore, the present study demonstrated that prejudiced beliefs might function as a buffer against gaining knowledge about the mental health help care system. Younger students and males were more prejudiced and had less knowledge.

### Effects of intervention upon mental health literacy

Identification of symptom profiles changed contingent on the intervention, and most probably on the information delivered during the three-day school programme. Knowledge increased substantially for anxiety, depression and schizophrenia, and moderately for anorexia. However, anxiety and depression are the by far the most prevalent disorders among adolescents [3]. Our data show a need for knowledge about internalising disorders among adolescents, and that it can relatively easily be met. Still, adolescents also need to know more about schizophrenia and other psychoses, not the least because sufferers from psychoses are more often the victims of public stigma than sufferers from internalisation disorders.

Anxiety disorder, one of the most prevalent mental disorders among adolescents, was the least known, identified by only one in ten adolescents. Depression, the most prevalent mental disorder in the general population, was only identified by one third of the adolescents. Interestingly, the symptoms of psychosis (schizophrenia) were almost as easily identified as those of depression. Anorexia nervosa was recognized by a majority of the adolescents.

Both depression and schizophrenia were identified by a smaller proportion in our sample of Norwegian adolescents than by young adult Australians [16]. One reason for this may be that in our study only a list of cues was presented, and not a more detailed case vignette, as in the Australian study. Another reason may be that the Australian sample was older, and thus probably more experienced and knowledgeable, than the youths in the present study. A third explanation may be that the level of mental health literacy is higher in Australia than in Norway, due to information campaigns, aimed for mental health literacy and early detection of mental disorder [36]. It is however noteworthy that symptom recognition increased equally for the depression and the schizophrenia profile, after intervention.

Anxiety disorders and anorexia nervosa were the least and most recognized profiles in our study. Almost similar low identification of anxiety disorder was reported in a US sample [17]. It is a paradox that one of the most prevalent disorders in youths, anxiety, is so poorly recognized, while the rare disorder anorexia is easily recognized. The explanation may be found in youth culture and media focus. Anorexia is a visible disorder, attracting much media attention, and can be associated with actors, pop stars, and athletes. On the other hand, few films or TV series with an adolescent audience have a leading character suffering from an anxiety disorder.

Knowledge about symptoms and mental health issues in general may be seen as cognitive components of mental health literacy, while prejudiced beliefs in addition have a negative affective component. The relationship between these cognitive and affective components of the mental health literacy construct is not necessarily linear. Interestingly, this study has demonstrated that prejudiced beliefs did decline in those who were exposed to a three-day universal school intervention. Whether the decline is related to the information given during the intervention, or to the different individual, group and plenary assignments in the three-day intervention, is a question left over for future research. However, this study demonstrates that adolescents prejudiced beliefs about mental health problems can be changed.

The present study demonstrated that there was a shift from mentioning the specialist health care system as a place to seek help, towards mentioning the primary health care system as the preferred place to seek help. There was also a tendency of increased mentioning of help at home, or self-help after intervention. Since the primary health care system is the gatekeeper to the specialist health care system in Norway, these shifts are adequate, since they are in direction of a more effective path to receiving help. Furthermore, Norwegian youths are dependent on their parents to receive specialist health care, since they are minors in health care until they are 16 years old. Thus seeking help at home for the youngest subjects is adequate behaviour.

### The impact of gender in mental health literacy

Gender had significant impact on both symptom profile recognition and prejudiced beliefs, and, to a lesser degree, on knowledge about places to seek help. The sex difference in recognition of depression reflected the difference found in young Australians [16]. Girls were also better than boys in identifying anxiety disorders and anorexia nervosa. Similar as in Australian young adults [16], no gender difference was found in the identification of schizophrenia in Norwegian youths. We have no cues in the data to explain these gender differences, so what follows is pure speculation. However, anxiety, depression and anorexia nervosa are more prevalent in women, while there is no sex difference in the prevalence of schizophrenia [37]. Perhaps young girls and boys have “gendered” awareness of the disorders they are at risk for themselves.

Being a girl predicted lower level of prejudiced beliefs about mental disorders in our sample. Previous research has also demonstrated that females are less prejudiced towards mental illness [9,38].

Finally, gender had less impact on knowledge about where to seek help. Only the mentioning of primary health care was influenced by respondent’s female sex. We know from a previous study of the North Norwegian youth population [27] that girls utilize the primary health care system more and that the specialist mental health services are equally utilized by both sexes.

### The importance of age

Some small, but important, age differences were found in the present study. For symptom profile recognition, 10th grades (mean age 15 years) knew more than 8th graders (mean age 13) and the age difference in knowledge increased after the intervention. Whether this could be read as an interaction effect was however not confirmed. However, this may indicate that the younger adolescents are not yet cognitively ready to absorb and understand specific knowledge about symptom profiles and syndromes.

Furthermore, lower grade students held more prejudiced beliefs than higher grade students. Prejudiced and stereotypical beliefs may be viewed as correlates of identity development statuses in adolescence, in accordance with Kroger and Marcia’s [39] theories, and stereotypes are indicators of less mature identity statuses.

Finally, we found that increasing age was a predictor of mentioning primary health care as a place to find help. Since the older adolescents in this study were reaching the age for being autonomous users of health services, according to Norwegian legislation, this finding indicates that increased awareness of easily available health care follows increasing age in our sample.

### Prejudice as a buffer against knowledge

According to Thornicroft and colleagues [23, p 192], *“The main challenge in the future is to identify which interventions will produce behaviour change to reduce discrimination against people with mental illness.”* Earlier studies of school programmes aimed at reducing stigma have remained inconclusive [40]. In the present study, we have identified a direct path between prejudiced beliefs and the lack of knowledge about places to seek help for mental health problems. Endorsing more prejudiced beliefs was directly related to not mentioning places for help. Furthermore the school intervention had no effect in changing *No places mentioned* into suggesting a place to seek help. Perhaps prejudice worked as a kind of “insulation” or buffer against gaining knowledge?

In the present study we have demonstrated that youths with prejudiced beliefs about mental health problems were difficult to reach with this specific universal mental health school program, since many of them remained ignorant about the help system after intervention. We do not know from our data whether the lack of knowledge would remain if the subjects themselves experienced a need for help. However, Barney and colleagues [29] demonstrated that subjects with depressive symptoms reported feelings of embarrassment about seeking professional help, and negative beliefs about other people’s reactions to them; and that this self-stigmatization was related to lower probability of help-seeking from professional sources. It is reasonable to fear that prejudice both may serve as a hindrance against seeking help for one-self, and also against recognizing problems and seeking adequate help for those who may be the future dependents of the prejudiced person. Thus it is doubly important to fight stigma, both self-stigma and stigma aimed at mentally ill.

### Strengths and limitations

The strengths of this study were the sample size, the high adherence of participants at pre-test and follow-up and that the mental health literacy construct has been operationalized into measurable units with good face validity.

The written symptom profiles in the present study were very short, only presenting a list of symptoms. Other studies have made vignettes in the form of short stories [12,17]. There is variety of form, detail and quality of vignettes for case identification in current research, and there is need for standardisation and validation of this method for measuring mental health literacy.

The measurement of prejudiced beliefs is complicated, since the construct is complex. The internal consistency of the prejudiced belief scale was marginal at pre-test. However, the stability of the scale improved at post-test. Furthermore, a factor analysis demonstrated that a single component explained the four items well. The content of the scale had high face validity, since it clearly contained utterances of prejudiced beliefs about mental health issues. Furthermore, the scale gave valuable information and seemed to be a key variable in the study of mental health literacy in the present sample. As lower reliability implies lower statistical power, the true intervention effect on prejudices in the population would be expected higher given an improved internal consistency.

Categorizing answers to open-ended questions is challenging. In classifying suggestions of places to find help, three categories were rather clear-cut: “no answer”, “primary health care” and “specialist health care”. The fourth and smallest category “home, self-help, internet” covers help-seeking from non-professional sources, or from written sources, and is a somewhat heterogeneous, “other answers”, category. If splitting in smaller more homogenous categories, statistical power would have been lost. Another issue is that we measured health service knowledge through mentioning of places, and did not ask about help seeking behaviour in terms of actual use.

There were significant differences in mental health literacy between the intervention and the control group at pre-test which may offer an alternative hypothesis as to the origin of change from pre-test to follow-up. However, we employed statistical methods that could separate the effect of group differences from the intervention. We offer two possible explanations for the baseline differences. Firstly, there could be socio-economic differences between the uptake area of the intervention school and the two control schools. Regretfully, we did not ask for parent’s length of education, which could be used to control for differences in socio-economic status between intervention and control group. We did however obtain information about school average academic scores on national standardized tests three years after the intervention, and all schools scored slightly above average and had rather similar scores, so we have no indication of major academic differences between the three schools. Secondly, since the students had received information about the research project, since their teachers had been primed, and since the school schedule was announcing the three day intervention beforehand, students at the intervention school knew that they were about to engage in the three day educational programme when they completed the pre-test. Thus, it is possible that this may have resulted in priming effects. However, despite these baseline differences, the intervention seems to have had an independent effect.

An alternative design was also considered, splitting all three schools into an intervention and a control group. However, since the implementation of the programme required that three consecutive school days were assigned to mental health issues, we suspected that a considerable leakage of information would occur from the intervention group to the control group, since friendship and extra-curricular activities happen across school class-boundaries. It has been shown that to be successful, universal programmes should be implemented in the entire school, by changing the curriculum of all classes [5].

A possible confounding variable to the results about identification of symptom profiles may be that the specific information about mental disorders was taught in a uniform manner by three graduate students of clinical psychology to the majority of classes. The remaining class teachers considered themselves knowledgeable about the specific mental disorders and performed this lecture themselves, after briefing. We did however not register teacher vs researcher as a variable, or any other information about the teaching skills, or programme adherence of the teachers, and could thus not control for these possible confounders. It has been shown that teacher related factors, such as teaching skills and programme adherence are predictors for success for school programmes for mental health [5]. Thus giving the teachers the choice to teach the specific knowledge about mental health themselves, or having it delivered by an expert, may perhaps have ensured that these issues were taught in an optimal manner in all classes. Most school have school nurses, psychologists and counsellors with special competence in mental health issues. These should be actively involved in universal mental health promotion programmes. One should however not overrate the possible impact of this single lesson, since the class teachers were in charge of the remaining mental health promoting activities during three consecutive school days.

The follow-up was performed three month after the intervention, so long-term effects and possible decay of effect of the programme was not reported here.

### Implications

We found systematic sex effects for mental health literacy, and gender specific programmes for enhancing mental health literacy in male adolescents should thus be considered. An equally important implication is that older adolescents had less prejudice and more knowledge. Thus perhaps the programme should be revised and made more age-adequate for the youngest students? School programmes should focus on enhancing knowledge about the common mental disorders, like anxiety disorders and depression, since we found that youths knew little about the most prevalent disorders. However, it is still important to impart balanced information about schizophrenia and other psychoses, since sufferers from these conditioned are often victims of prejudiced attitudes. Adolescents with prejudiced beliefs about mental disorders learnt less from this universal school intervention. For school programmes to be effective, prejudices should first be challenged, before imparting general information about mental health. Since younger adolescents are in need of knowledge about the health care system, to be able to take responsibility of their own health, it seems important that preventive programs impart information about the system and where to seek help. Health services should also be aware of adolescent’s need of information about available help and aim information about availability at these young users. Our findings speak for well-balanced information programmes about mental disorders among youths, challenging prejudice against mental disorders and focusing more on anxiety and depression.

## Conclusions

The low cost universal school program had effect on both recognition of mental disorders, prejudice and knowledge about where to seek help, and consequently on mental health literacy of adolescents. Prejudice appears to be closely knit to knowledge about places to seek help. These findings shed light upon the relationship between prejudice, stigma and possible self-stigma that hinders adequate help seeking, both for one-self and possibly for others.

## Competing interests

The data collection was financed by a grant of NOK 200.000 from The Extra Foundation: Health and Rehabilitation (Extrastiftelsen Helse og Rehabilitering) through the member organisation THealth (Rådet for psykisk helse), which also is the publisher of “Mental health for everyone”. Other than financing the trial, and participating in the discussion of prejudice about mental health items, The Norwegian Council for Mental Health had no role in and no influence on the research presented in this paper.

## Authors’ contributions

IS, CEAW, CB, LIJ and YA had the idea and designed the study. CB, LIJ and YA organized the intervention and performed the data collection. IS and OF analysed the data. IS drafted the manuscript. CB, LIJ and YA contributed in drafting the description of procedures and OF contributed in drafting the statistics and results sections. OF and CEAW participated in the critical revision of the manuscript. All authors have read and approved the final manuscript.

## Pre-publication history

The pre-publication history for this paper can be accessed here:

http://www.biomedcentral.com/1471-2458/13/873/prepub
